# Intestinal Microbiome Richness of Coral Reef Damselfishes (*Actinopterygii: Pomacentridae*)

**DOI:** 10.1093/iob/obac026

**Published:** 2022-09-16

**Authors:** Christopher R J Kavazos, Francesco Ricci, William Leggat, Jordan M Casey, J Howard Choat, Tracy D Ainsworth

**Affiliations:** Biological, Earth and Environmental Sciences, The University of New South Wales, Kensington, NSW 2052, Australia; Biological, Earth and Environmental Sciences, The University of New South Wales, Kensington, NSW 2052, Australia; Centre of Marine Bio-Innovation, The University of New South Wales, Kensington, NSW 2052, Australia; School of Environmental and Life Sciences, The University of Newcastle, 10 Chittaway Dr, Ourimbah, NSW 2258, Australia; Australian Research Council Centre of Excellence for Coral Reef Studies, James Cook University, Townsville, QLD 4811, Australia; PSL Université Paris: EPHE-UPVD-CNRS, USR 3278 CRIOBE, Université de Perpignan, Perpignan 66100, France; Laboratoire d'Excellence “CORAIL,” Université de Perpignan, Perpignan 66100, France; College of Science and Engineering, James Cook University, Townsville QLD 4814, Australia; Biological, Earth and Environmental Sciences, The University of New South Wales, Kensington, NSW 2052, Australia; Centre of Marine Bio-Innovation, The University of New South Wales, Kensington, NSW 2052, Australia

## Abstract

Fish gastro-intestinal system harbors diverse microbiomes that affect the host's digestion, nutrition, and immunity. Despite the great taxonomic diversity of fish, little is understood about fish microbiome and the factors that determine its structure and composition. Damselfish are important coral reef species that play pivotal roles in determining algae and coral population structures of reefs. Broadly, damselfish belong to either of two trophic guilds based on whether they are planktivorous or algae-farming. In this study, we used 16S rRNA gene sequencing to investigate the intestinal microbiome of 5 planktivorous and 5 algae-farming damselfish species (*Pomacentridae*) from the Great Barrier Reef. We detected *Gammaproteobacteria* ASVs belonging to the genus *Actinobacillus* in 80% of sampled individuals across the 2 trophic guilds, thus, bacteria in this genus can be considered possible core members of pomacentrid microbiomes. Algae-farming damselfish had greater bacterial alpha-diversity, a more diverse core microbiome and shared 35 ± 22 ASVs, whereas planktivorous species shared 7 ± 3 ASVs. Our data also highlight differences in microbiomes associated with both trophic guilds. For instance, algae-farming damselfish were enriched in *Pasteurellaceae*, whilst planktivorous damselfish in *Vibrionaceae*. Finally, we show shifts in bacterial community composition along the intestines. ASVs associated with the classes *Bacteroidia, Clostridia*, and *Mollicutes* bacteria were predominant in the anterior intestinal regions while *Gammaproteobacteria* abundance was higher in the stomach. Our results suggest that the richness of the intestinal bacterial communities of damselfish reflects host species diet and trophic guild.

## Brazilian Portuguese

O sistema gastro-intestinal de peixes abriga microbiomas diversos que afetam a digestão, nutrição e imunidade do hospedeiro. Apesar da grande diversidade taxonômica dos peixes, entende-se pouco sobre o microbioma dos peixes e fatores que determinam sua estrutura e composição. Peixes-donzela são espécies importantes em recifes de coral que exercem papéis pivotais na determinação da estrutura de algas e corais dos recifes. De forma geral, peixes-donzela pertencem à uma de duas guildas tróficas dependendo se são planctívoros ou algívoros. Nesse estudo, usamos sequenciamento do gene 16S rRNA para investigar o microbioma intestinal de cinco espécies planctívoras e cinco espécies algívoras de peixes-donzela (Pomacentridae) da Grande Barreira de Corais. Detectamos ASVs de *Gammaproteobacteria* pertencendo ao gênero *Actinobacillus* em 80% dos indivíduos amostrados nas duas guildas tróficas, logo, bactérias desse gênero podem ser consideradas como possíveis membros essenciais do microbioma dos pomacentrídeos. Peixes-donzela algívoros apresentaram uma maior alpha-diversidade bacteriana, um microbioma essencial mais diverso e compartilharam 35 ± 22 ASVs, e espécies planctívoras compartilharam 7 ± 3 ASVs. Nossos dados também ilustram diferenças nos microbiomas associados com ambas guildas tróficas. Por exemplo, peixes-donzela algívoros estavam enriquecidos em *Pasteurellaceae*, enquanto peixes-donzela planctívoros, em *Vibrionaceae*. Finalmente, demonstramos mudanças na composição da comunidade bacteriana associada com as classes *Bacteroidia, Clostridia* e *Mollicutes* foram predominantes nas regiões intestinais anteriores enquanto a abundância de *Gammaproteobacteria* foi maior no estômago. Nossos resultados sugerem que a riqueza das comunidades bacterianas intestinais de peixes-donzela refletem a dieta da espécie do hospedeiro, bem como a sua guilda trófica.

## Chinese

鱼类肠道中种类丰富的微生物菌群对于鱼类的消化、营养和免疫都有影响。尽管鱼类的分类多样性很高, 但我们对于鱼类体内的微生物菌群及能够影响其结构和组成的因素却知之甚少。雀鲷科鱼类是一种重要的珊瑚礁鱼类, 并且对珊瑚礁中的藻类和珊瑚种群结构起到关键性作用。概括来说, 基于食性的不同(以浮游生物为食或以藻类为食), 雀鲷科鱼类分属于两种摄食类群。在本研究中, 我们利用16S rRNA基因序列对于大堡礁的五种以浮游生物为食的雀鲷和五种以藻类为食的雀鲷分别进行了研究。在这两种类群80%的样本中, 我们都发现了属于放线杆菌属*Actinobacillus*的Gammaproteobacteria的扩增子序列变体。因此, 此属细菌很可能是雀鲷肠道微生物菌群的主要组成。食藻类雀鲷具有更高的细菌α多样性, 它们的核心微生物菌群的多样性更高, 共享了35 ± 22个扩增子序列变体, 而食浮游生物类雀鲷的核心微生物菌群则只共享了7 ± 3个扩增子序列变体。我们的数据还突出了两种营养类群肠道微生物的区别。例如, 食藻类雀鲷有更多的*Pasteurellaceae*, 而食浮游生物类雀鲷则有更多的*Vibrionaceae*。最后, 我们还展示了肠道中细菌群落的更替。在肠道前部, *Bacteroidia, Clostridia*和*Mollicutes*占据主导地位;而在胃中, *Gammaproteobacteria*则丰度更高。我们的结果意味着肠道菌群的丰富性反映了鱼类宿主的食性和摄食类群。

## Hindi

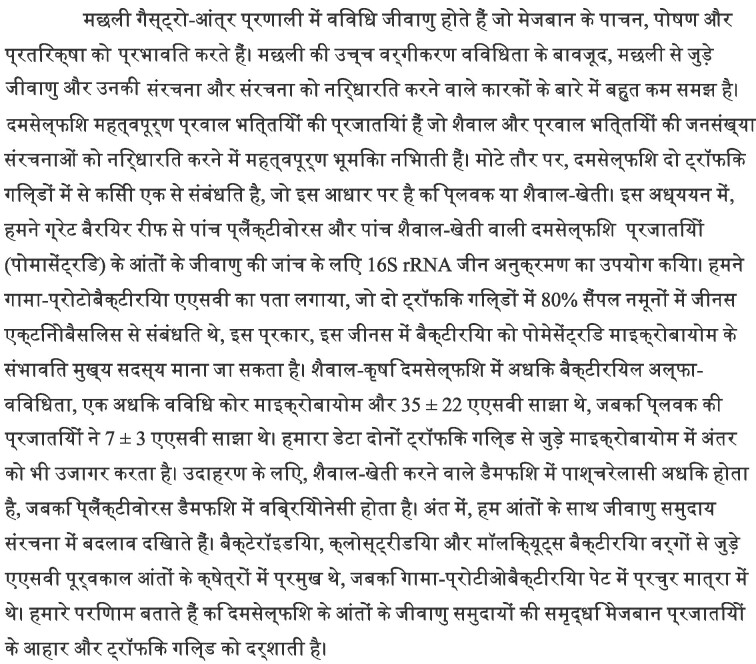

## Italian

Il sistema gastro intestinale dei pesci ospita un microbiota che influenza la digestione, nutrizione e sistema immunitario dell'ospite. Nonostante l'enorme diversità taxonomica dei pesci, la nostra comprensione del microbiota di questi animali ed i fattori che determinano la sua struttura e composizione è ancora scarsa. I pesci damigella includono specie importanti per le barriere coralline che forniscono servizi in grado che influenzare la struttura delle popolazioni di alghe e coralli. In generale, i pesci damigella appartengono a due gruppi funzionali basati sul loro tipo di dieta, e vengono divisi in consumatori di plankton o alghe. In questo studio abbiamo sequenziato il gene 16S rRNA per investigare il microbiota intestinale di cinque pesci damigella (Pomacentridae) che consumano plankton e cinque che consumano alghe provenienti dalla Grande Barriera Corallina. Abbiamo rilevato che l’80% degli individui analizzati in entrambi i gruppi funzionali avevano ASVs di *Actinobacillus* appartenenti al phylum dei *Gammaproteobatteri*, così, suggeriamo che batteri appartenenti a questo genere possono essere considerati membri essenziali del microbiota dei Pomacentridi. I pesci damigella che consumano alghe avevano una maggiore diversità (alpha), un microbiota essenziale più vasto e condividevano 35 ± 22 ASVs, mentre le specie che consumano plankton condividevano 7 ± 3 ASVs. I nostri dati evidenziano differenze nel microbiota associato con pesci appartenenti ai due gruppi funzionali. Per esempio, pesci damigella che consumano alghe avevo un maggior numero di ASVs di *Pasteurellaceae*, mentre le specie che consumano plankton avevano più *Vibrionaceae*. In fine, riportiamo variazioni nella composizione delle comunità batteriche lungo l'intestino. ASVs appartenenti alle classi batteriche *Bacteroidia, Clostridia* e *Mollicutes* erano più abbondanti nell'intestino anteriore mentre i *Gammaproteobacteria* nello stomaco. I nostri resultati suggeriscono che la diversità delle comunità batteriche dell'intestino dei pesci damigella riflette la dieta ed il gruppo funzionale dell'ospite.

## Background

Fishes represent the greatest taxonomic diversity of vertebrates, and despite our understanding of the importance of intestinal microbiota of terrestrial vertebrates, we still lack an understanding of fish microbiome diversity and functioning (Clements et al. 2014). Largely, fish microbiome studies have centered around species with commercial value, including trout, salmon, and carp (Wang et al. 2018). For example, gastrointestinal fish microbiomes are known to be important in intestinal cell proliferation (Rawls et al. 2004; Cheesman et al. 2011), nutrition ( Ray et al. 2012; Clements et al. 2014), and immunity (Bates et al. 2006; Bates et al. 2007; Galindo-Villegas et al. 2012). These studies show that the intestines of fishes harbor a large abundance and diversity of bacteria (Nayak 2010) and the regulation of this diversity is important in the maintenance of host health through a complex set of microbe-microbe and microbe-host interactions ( Neish 2009; Foster et al. 2017).

There are many factors that affect the structure of fish gastrointestinal microbiomes (Clements et al. 2014; Wang et al. 2018). These include host-related factors such as genetic attributes, size, age, sex (Bolnick et al. 2014; Li et al. 2016; Stephens et al. 2016), host phylogeny (Sullam et al. 2012; Li et al. 2014; Miyake et al. 2015), environmental factors (such as water quality) (Hagi et al. 2004; Sullam et al. 2012; Neuman et al. 2016), and host diet (Miyake et al. 2015; Neuman et al. 2016). Studies that investigated intestinal microbiome changes have mostly focused on the impact of fish foods on species of aquaculture importance (Ringø et al. 2006; Martin-Antonio et al. 2007), although a few studies have investigated wild fish populations (Miyake et al. 2015; Zhang et al. 2018). For instance, bacterial symbionts diversification in wild herbivorous surgeonfish intestines is thought to be an important driver of host niche-partitioning (Miyake et al. 2016; Ngugi et al. 2017), suggesting that intestinal microbiomes can influence the trophic ecology of coral reefs and facilitate resource partitioning in these hyper-diverse ecosystems. However, the involvement of intestinal bacteria in wild fish physiology remains largely unknown.

There is increasing evidence that herbivorous fishes have distinct microbiomes as compared to omnivorous and carnivorous fishes (Givens et al. 2015). Herbivorous and carnivorous diets are known to cause shifts in intestinal fish microbiomes; fishes with plant-based diets have intestinal microbiomes dominated by *Firmicutes*, such as *Clostridium*, while fishes with fat-based diets have microbiomes dominated by protease-producing *Proteobacteria* (Desai et al. 2012; Ingerslev et al. 2014; Liu et al. 2016). In addition, the diversity of herbivorous fish intestinal microbiomes is higher than omnivorous and carnivorous host species under similar environmental conditions (He et al. 2013), suggesting that host feeding behavior has a significant effect on fish intestinal microbiomes.

Damselfishes (*Pomacentridae*) are a diverse and abundant group of coral reef fishes (Cooper et al. 2009; Campbell et al. 2018), and they are among the most widely studied families (Choat 1991; Emslie et al. 2019). Broadly, damselfishes are grouped into either planktivorous or algae-farming trophic guilds, although some herbivorous species may also feed on zooplankton (Eurich et al. 2019). Planktivorous damselfishes play a key role in transferring energy from the plankton to higher tiers of the food chain, while algae-farming damselfishes influence sediment and algae dynamics on coral reefs and may increase the presence of coral disease-associated pathogens within their territories (Casey et al. 2015; Casey et al. 2015; Emslie et al. 2019; Randazzo Eisemann et al. 2019, Tebbett et al. 2020; Blanchette et al. 2019). Algae-farming species can be differentiated based on the algal composition within their territories, and they are divided into several behavioral guilds, including indeterminate grazers, extensive grazers, and intensive grazers (Hata and Kato 2004; Emslie et al. 2012; Casey et al. 2015; Emslie et al. 2019). Indeterminate and extensive grazers feed both on macroalgae and turf, while intensive grazers maintain distinct areas of turf algae through selective grazing and weeding of unpalatable algae (Gibson et al. 2001; Emslie et al. 2012). Intensive grazing damselfish are also referred to as algae farmers. Research on intensive grazers has focused on competition (Eurich et al. 2018), patterns of co-existence (Eurich et al. 2018; Eurich et al. 2018; Eurich et al. 2019), behavioral interactions (Kasumyan 2009; Weimann et al. 2018), and their role in structuring algae and coral communities (Klumpp et al. 1987; Ceccarelli et al. 2005; Ceccarelli 2007; Gochfeld 2010; Casey et al. 2014; Casey et al. 2015).

In this study, we investigated and described the intestinal microbial diversity of ten species of planktivorous and algae-farming damselfishes, two guilds that significantly impact coral reef trophic dynamics. We hypothesized that differences in intestinal microbial communities will reflect the differences between these two trophic guilds. Specifically, across the different host species and trophic guilds, we examined (1) differences in bacterial communities across fish species and trophic guilds, (2) core microbial members, and (3) changes in microbial community structure along the length of the intestinal tract.

## Methods

### Species collections and dissections

Fishes were collected from the Heron Island lagoon in the southern Great Barrier Reef, Australia (23°26′53″S, 151°56′52″E) in January and February 2015. Collections occurred at a depth of 1–8 m adjacent to the Heron Island Research Station. Three individuals of ten sympatric damselfish species (*Abudefduf sexfasciatus, A.whitleyi, Acanthochromis polyacanthus, A. polyacanthus, Chromis atripectoralis, Dischistodus pseudochrysopoecilus, D. perspicillatus, Pomacentrus moluccensis, P. wardi, Stegastes apicalis*, and *S. nigricans*) of similar lengths were randomly collected across the two trophic guilds planktivorous and algae-farming. Each trophic guild was represented by 5 species and 15 individuals. Collections were conducted on SCUBA, and the planktivorous species were collected using a barrier net, while the algae-farming species were collected using a speargun. Following collections, the fishes were immediately placed on ice and transported to Heron Island Research Station. In the laboratory under sterile conditions, fishes were weighed, measured and photographed, then the gastrointestinal tract was removed, and the gut length was recorded and photographed. The entire gut was fixed in 4% DNA/RNA free paraformaldehyde and sterile phosphate-buffered saline for 12 h, then it was stored in DNA/RNA free water.

### DNA extraction, amplification, and sequencing

Samples were transported to James Cook University for subsampling along each intestinal tract and DNA extraction. Under sterile conditions, standardized biopsy cores (3 × 3 mm) were taken from four locations along the intestinal tract: the stomach, the anterior intestine, the mid-intestine, and the posterior intestine. DNA was extracted from tissue biopsies using a QIAamp DNA Micro Kit (Qiagen, Hilden, Germany) following the manufacture's guidelines. A nanodrop was used to record the quality (260/280 ratio) and quantity (ng/μL) of DNA from each extraction.

Amplification of the 16S V1-V3 rRNA gene region was done using the primers 27F (5′-AGRGTTTGATCMTGGCTCAG-3′) (Ludwig 2007) and 519R (5′-GTNTTACNGCGGCKGCTG-3′) (Lane et al. 1985) with barcodes on the forward primer. These 16S rRNA genes were amplified using the HotStarTaq Plus Master Mix Kit (Qiagen, USA) under the following conditions: 94°C for 3 min, followed by 28 cycles of 94°C for 30 s, 53°C for 40 s and 72°C for 1 min, after which a final elongation step at 72°C for 5 min was performed. After amplification, PCR products were checked in 2% agarose gel to determine the success of amplification and the relative intensity of bands. Multiple samples were pooled together (e.g., 100 samples) in equal proportions based on their molecular weight and DNA concentrations. Pooled samples were purified using calibrated Ampure XP beads. Then the pooled and purified PCR products were used to prepare a DNA library by following Illumina TruSeq DNA library preparation protocol. Sequencing was performed at the Molecular Research LP (MR DNA; Texas, USA) on a MiSeq V2 System following the manufacturer's guidelines.

Amplicon sequence data were sorted by the sample and demultiplexed using *demux* for QIIME 2 (version 2018.11; (Bolyen et al. 2018.)). Sequences were screened for quality, trimmed at 450 bp after removal of primer sequences, and assigned as amplicon sequence variants (ASVs) using DADA2 (Callahan et al. 2016). Taxonomy of the ASVs was determined using a pre-trained, naїve Bayes classifier (Pedregosa F) and the q2-feature-classifier plugin (Bokulich et al. 2018). The classifier was trained on the target 480 bp region of sequences in the Greengenes 13_8 99% database. ASV clusters were arranged in a phylogenetic tree using FastTree (Price et al. 2010) and visualized using Interactive Tree of Life 3.6.1 (Letunic and Bork 2016). The feature table, metadata, and taxonomic classifications were exported from QIIME 2 in .biom format and the rooted phylogenetic tree was exported in .nwk format. The closest known sequences and the origin of selected ASVs were identified through a BLASTN-based search against the GenBank nr/nt database.

### Statistical analysis

The feature table and phylogenetic tree were imported into R version 3.5.2 and stored as a *phyloseq* object (McMurdie and Holmes 2013) for downstream analyses. All ASVs not assigned to phylum were filtered from the data, and those designated as chloroplasts or cyanobacteria were removed and stored as a separate object for further analysis. Samples were rarefied to minimum sampling depth for alpha-diversity analyses, which was estimated using the R package *vegan* (Oksanen et al. 2017). Non-rarefied data were used for generalized linear model (GLM) analysis (McMurdie and Holmes 2014; McMurdie 2018). Data used for principal component analysis (PCA), betadisper-test and PERMANOVA were computed using centered log-transformed Euclidean distance matrices of the non-rarefied ASV table. Differences in alpha-diversity between trophic guilds were tested via *t*-test. Multivariate GLM was used to test for significant differences in bacterial communities among host fish species, trophic guild, and location along intestines using *mvabund* in R (Wang et al. 2012). PCA, betadisper-test and PERMANOVA were used to test differences in the communities of *Proteobacteria, Bacteroidetes*, and *Firmicutes* among fish species and between the two trophic guilds. Bacterial taxa were grouped by class when examining microbiome changes along the length of the intestinal tract. Bacterial community data were fitted to negative binomial distributions and tested using log-likelihood ratios (LRT) via 999 simulations using Monte Carlo resampling. A nested analysis of variance (ANOVA) used to test the role of trophic guild and gut location when accounting for species variation. Venn diagrams were produced using the *VennDiagram* package (Chen and Boutros 2011).

## Results

A total of 1,254,909 sequences were detected in 119 samples after denoising and removing all chloroplast, mitochondria, and uncharacterized sequences. Among these sequences, 3,776 ASVs were detected; 39.4% of which belonged to the phyla *Proteobacteria*, 26.2% to *Bacteroidetes*, 13.4% to *Firmicutes*, and 12.6% to *Planctomycetes*. The 20 most abundant ASVs accounted for 41% of the total number of detected sequences. The most common ASV belonged to the genus *Actinobacillus* and accounted for 9.9% of the total detected sequences (Table 1). Two unknown species of *Mollicutes* and *Pasteurellacea* accounted for 6.9 and 3.8% of sequences, respectively.

**Table 1:** Sequence abundance and taxonomy for each ASVs representing more than 1% of total sequences. Accession numbers for closest GenBank sequences (similarity given in brackets) are supplied.

ASV	Phylum	Lowest taxonomic division	Number of sequences	Proportion of total (%)	GenBank accession number
b727	*Proteobacteria*	*Actinobacillus sp.*	124,499	9.9	KT952745 (97.5%)
5647	*Tenericutes*	*Mollicutes*	87,057	6.9	HG971018 (96.3%)
94ba	*Proteobacteria*	*Pasteurellacea*	47,527	3.8	KT952745 (93.5%)
3023	*Firmicutes*	*Ruminococcaceae*	26,355	2.1	MG488771 (98.8%)
6350	*Tenericutes*	*Mycoplasmataceae*	24,219	1.9	LN612674 (91.5%)
9b2f	*Proteobacteria*	*Pasteurellacea*	24,219	1.9	KT952745 (91.9%)
d532	*Proteobacteria*	*Alteromonadales*	23,877	1.9	KT952746 (100.0%)
5a8a	*Proteobacteria*	*Vibrio ponticus*	22,112	1.8	MG524941 (100%)
7936	*Proteobacteria*	*Alteromonadales*	15,147	1.2	KT952746 (99.8%)
596f	*Proteobacteria*	*Gammaproteobacteria*	14,436	1.2	LC121875 (88.4%)
73d1	*Proteobacteria*	Vibrio sp.	13,977	1.1	KT952854 (98.7%)
6013	*Proteobacteria*	*Pasteurellacea*	13,435	1.1	KT952745 (92.3%)
af86	*Firmicutes*	*Clostridium colinum*	13,177	1.1	KC993540 (94.2%)

**Fig. 1:**
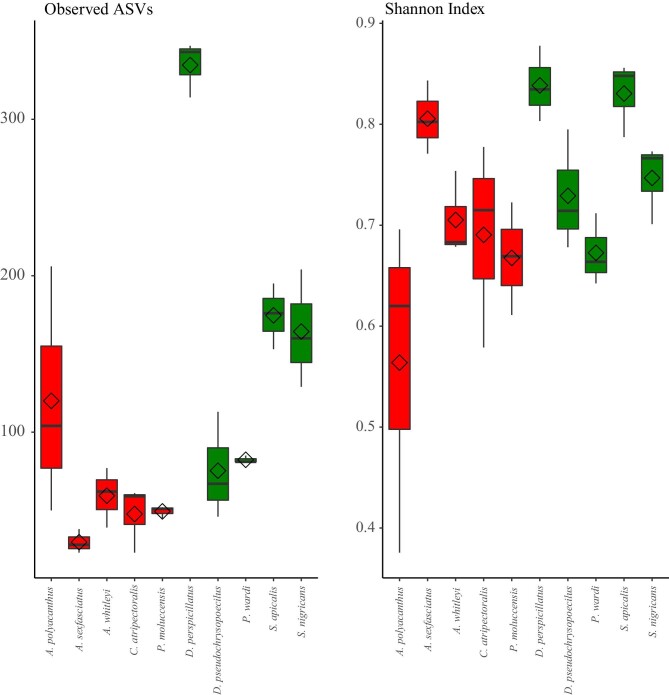
Observed richness and Shannon diversity for each fish species. Planktivorous host species are shaded red and algae-farming species are shaded green.

**Fig. 2:**
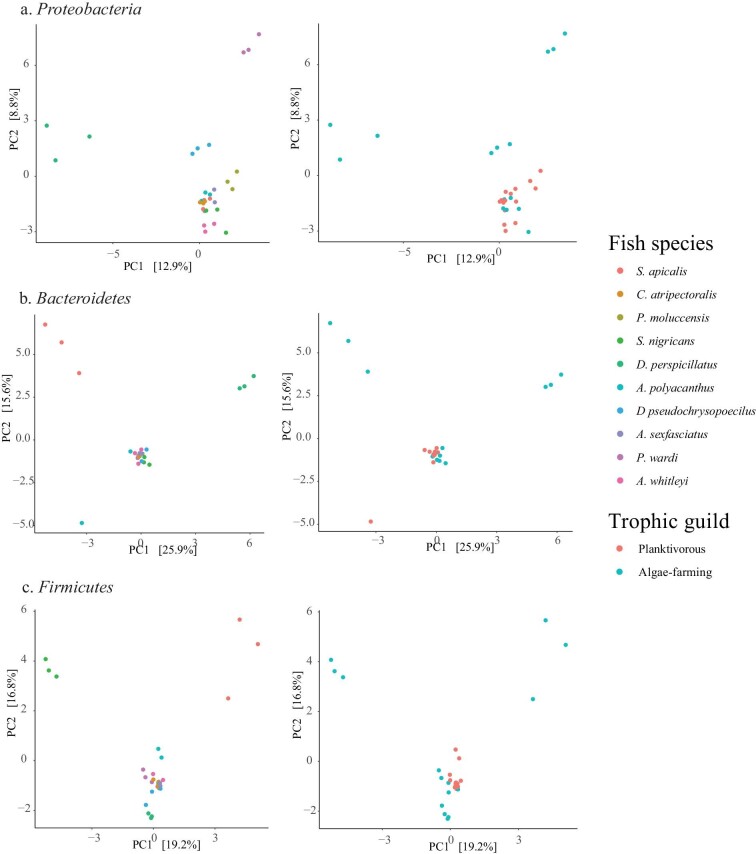
PCA biplots showing individual fish intestinal microbiomes for *Proteobacteria, Bacteroidetes*, and *Firmicutes*. Ordinations are divided by fish species (left) and trophic guild (right).

Different ASV richness was detected for each fish species with observed ASVs (t = −3.15, *P* = <0.01) and Shannon index (t = −3.68, *P* = <0.01) differing significantly between the two trophic guilds. The damselfish *D. perspicillatus* had the greatest mean richness of ASVs, with a total of 322 ± 17 ASVs per individual (Fig. 1). The species with the lowest ASV richness were *C. atripectoralis* and *A. sexfasciatus* with 47 ± 21 and 30 ± 8 ASVs per individual, respectively (Fig. 1). Shannon diversity was greatest for two algae-farming species *D. perspicillatus* and *S. apicalis* and lowest for the planktivorous species *A. polyacanthus* and *P. moluccensis.* PCA biplots, betadisper-test, and PERMANOVA revealed that the beta-diversity of *Proteobacteria, Bacteroidetes*, and *Firmicutes* communities differed among fish species and trophic guilds (Fig. 2; Table 2).

**Table 2:** Results of betadisper-test and PERMANOVA testing the beta-diversity of Proteobacteria, Bacteroidetes, and Firmicutes communitites across fish species and between trophic guilds.

	Fish species		Trophic guild	
	betadisper	PERMANOVA	betadisper	PERMANOVA
*Proteobacteria*	*P = 0.084*	F = 1.86; p = 0.001***	p = 0.039*	F = 3.52; p = 0.001***
*Bacteroidetes*	*P = 0.269*	F = 1.78; p = 0.001***	*p = 0.233*	F = 2.41; p = 0.001***
*Firmicutes*	P = 0.001***	F = 2.17; p = 0.001***	*p = 0.355*	F = 3.92; p = 0.001***

### Core microbiomes

**Fig. 3:**
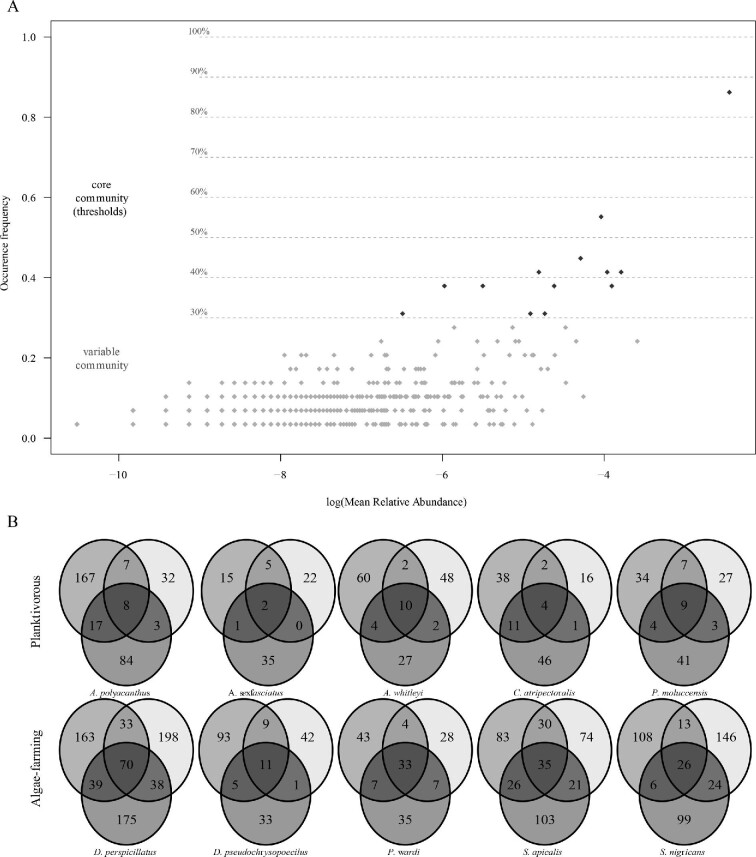
(**A**) Core members of the microbiome (blue) at different threshold levels. The variable community represents ASVs occurring in less than 30% of sampled individuals. (**B**) Venn diagrams depicting the number of ASVs shared between whole microbiomes of the three sampled individuals for each fish species. The top row represents planktivorous species and bottom row represent algae-farming species.

In line with previous studies that investigated the core microbiome of other organisms (Ainsworth et al. 2015; Ricci et al. 2022), we choose a minimum threshold of 30% for this metric. Most ASVs occurred in less than 30% of sampled individuals across all fish species (Fig. 3a). A total of 13 bacterial ASVs were found in more than 30% of sampled individuals; therefore, they may represent the 30% core microbiome of pomacentrid investigated in this study (Table 3). The most common ASV in this study belonged to the genus *Actinobacillus*, which occurred in more than 80% of sampled individuals (Table 3), albeit at a low abundance in many individuals, with the highest abundances in the planktivorous damselfishes *A. polyacanthus* and *P. moluccensis*.

**Table 3:** Taxonomic composition of core ASVs occurring in more than 80% of sampled individuals. Accession numbers for closest GenBank sequences (similarity given in brackets) are supplied. Occurrence and relative abundances were generated from rarefied data.

ASV	Phylum	Lowest taxonomic division	Occurrence (%)	Relative abundance	GenBank accession number
b727	*Proteobacteria*	*Actinobacillus sp.*	83.3	0.083	KT952745 (97.5%)
94ba	*Proteobacteria*	*Actinobacillus sp.*	53.3	0.017	KT952745 (93.5%)
9bd9	*Proteobacteria*	*Photobacterium damselae*	43.3	0.013	CP035457 (100%)
5647	*Tenericutes*	*Mollicutes*	40.0	0.022	HG971018 (96.3%)
a832	*Proteobacteria*	*Photobacterium damselae*	40.0	0.008	CP018297 (100%)
73d1	*Proteobacteria*	*Vibrio sp.*	40.0	0.010	KT952854 (98.7%)
9b2f	*Proteobacteria*	*Actinobacillus porcinus*	40.0	0.018	KT952745 (91.9%)
6c33	*Proteobacteria*	*Spirobacillales*	37.7	0.002	KU578602 (100%)
dc1c	*Proteobacteria*	*Vibrio sp.*	37.7	0.004	CP033144 (100%)
5a8a	*Proteobacteria*	*Vibrio ponticus*	37.7	0.019	MG524941 (100%)
762a	*Bacteroidetes*	*Lutimonas sp.*	30.0	0.001	MG488523 (99.6%)
ca47	*Proteobacteria*	*Vibrio harveyi*	30.0	0.009	CP033144 (100%)
6013	*Proteobacteria*	*Pasteurellaceae*	30.0	0.007	KT952745 (92.3%)

The core bacterial assemblages of each fish species (defined as ASVs that were shared between all sampled individuals for each species) were composed of a variable number of ASVs (Fig. 3b). For example, there were 70 bacterial ASVs shared between the three sampled individuals of *D. perspicillatus* and only two ASVs shared between the three *A. sexfasciatus* individuals. Core microbiomes within fish species were richer in algae-farming species than planktivorous species (Fig. 3b), with algae-farming species sharing 35 ± 22 ASVs and planktivorous species sharing only 7 ± 3 ASVs (Wilcox test W = 25, p < 0.01).

Core ASVs that occurred in all three individuals of a fish species belonged to the phyla *Bacteroidetes, Firmicutes, Tenericutes, Spirochaetes, Planctomycetes, Proteobacteria, and Verrucomicrobia*. Core ASVs belonging to *Coraliomargarita* sp. and *Verruco-5* (*Verrucomicrobia*), *Pirellulaceae* (*Planctomycetes*), and *Desulfovibrionaceae* (*Deltaproteobacteria*) occurred in all three sampled *D. perspicillatus* individuals (Supplementary Figure S1). We also detected high diversity of an unknown clade of *Gammaproteobacteria* in *P. moluccensis* and *P. wardi* damselfish. There were 61 core ASVs belonging to the *Bacteroidetes*, 28 of which occur in *S. apicalis* and 38 in *D. perspicillatus* (Supplementary Figure S2). An unknown clade of *Flavobacteriales* and a diverse consortium of *Rikenellaceae* were core members of *S. apicalis*, while *D. perpicillatus* had a diverse core assemblage of ASVs belonging to the family *Flavobacteriaceae*. One ASV belonging to *Spirochaetes, Brevinema andersonii*, was a core member of *S. nigricans* and *C. atripectoralis*, while a *Tenericutes* ASV belonging to Mollicutes was a core member of all fish species except the planktivorous damselfishes *A. polyacanthus* and *A. sexfasciatus* (Supplementary Figure S3). There was a rich consortium of core *Firmicutes* ASVs for *S. apicales* and *S. nigricans*, which included members of the *Erysipelotrichaceae, Ruminococcaceae*, and *Lachnospiraceae* families.

### Bacterial shifts along the intestinal tract

**Fig. 4:**
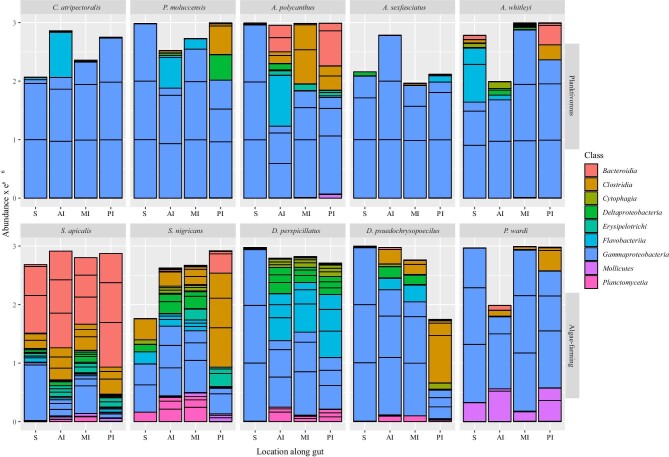
Changes in abundance of selected bacterial Classes along the four locations along the intestine of each species of damselfish as determined by nested multivariate generalized linear models. Intestinal locations include stomach (S), anterior intestine (AI), mid-intestine (MI), and the posterior intestine (PI). The top row represents planktivorous species and bottom row represent algae-farming species.

**Fig. 5:**
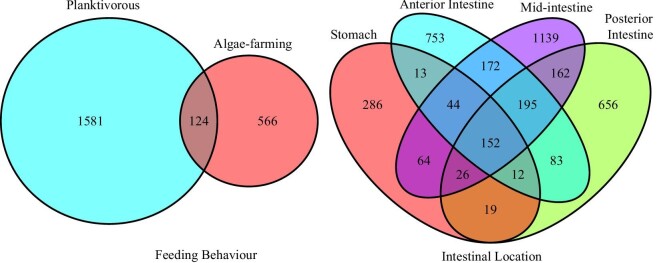
Venn diagrams depicting the number of shared ASVs for each trophic guild (left) and for each region of the intestine (right).

The interaction between the trophic guild and intestinal region had a significant influence on the gut bacterial community composition (LRT = 152, *P* = 0.001; Supplementary Table 1). The abundance of nine classes of bacteria changed significantly across the different fish species and locations along the intestinal tract (LRT = −0.0229, *P* < 0.001; Fig. 4; Supplementary Table 2). Members of *Gammaproteobacteria* were especially common throughout the planktivorous intestinal tracts, but we also found them along all the intestines regions of the algae-farming species *D. perspicillatus, D. pseudochrysopoecilus*, and *P. wardi* (Fig. 4). In intestinal regions where *Gammaproteobacteria* were uncommon, members of *Bacteroidia* and *Clostridia* were generally found at higher abundances—especially for algae-farming species (Fig. 4). Members of the *Mollicutes* and *Planctomycetia* were more common throughout the intestinal tracts of algae-farming hosts than planktivorous species although their abundances were generally lowest within the stomach region (Fig. 4). The stomach had 286 unique bacterial ASVs, the anterior intestine 753, while 1,139 and 656 ASVs were only found in the mid and posterior intestines, respectively (Fig. 5). Only 19 ASVs were common in the stomach and posterior intestine while 152 ASVs were found throughout the intestine (Fig. 5).

### Effect of the trophic guild on microbiomes

There was a significant difference in the microbiome composition between trophic guilds (LRT = −0.021, *P* < 0.001; Supplementary Table 2). Most bacterial ASVs were unique to either of the trophic guilds, with only 124 ASVs common to both guilds (Fig. 5). A total of 78 bacterial ASVs, belonging to 20 families, were important drivers of this relationship. There were marked differences in abundances of ASVs belonging to *Vibrionaceae, Lachnospiraceae*, and *Pasteurellaceae*. Two *Vibrio* sp. (*Vibrionaceae*) were more common in planktivorous species, and five ASVs of *Actinobacillus* (*Pasteurellaceae*) were more abundant in algae-farming species.

## Discussion

Our data show that algae-farming damselfish species have richer microbiomes than planktivorous species (Fig. 1) and this result is also reflected in their core bacterial community (Fig. 3). This result is likely attributable to the specialized feeding behavior of algae-farming species, which largely consume a narrow range of turf algae species (Hata and Kato 2004; Casey et al. 2014), unlike planktivorous species that are adapted to a more opportunistic feeding strategy. These results suggest that the microbiome structure of fish species with specialized feeding behavior has acquired specific intestinal bacteria and further research is needed to investigate how microbiome specialization affects host digestion and metabolism. We also note that other processes that were not tested in our study such as host phylogeny and functional traits could influence the composition of damselfish intestinal bacteria and ultimately influence fish physiology.

We found that similar to what was recorded in many other species of marine fish, the damselfish intestinal microbiome was dominated by members of *Proteobacteria, Bacteroidetes, Firmicutes*, and *Planctomycetes* (Table 1). For example, surgeonfish, parrotfish, and rabbitfish intestinal microbiomes from the Red Sea also consist of diverse assemblages of *Firmicutes* and *Proteobacteria* (Miyake et al. 2015). Another dominant ASV in the damselfish microbiome belonging to *Mollicutes* (*Tenericutes*) resembled bacteria detected in rabbitfish intestines (Zhang et al. 2018). The number of highly similar bacterial ASVs shared among pomacentrids, acanthurids, and siganids may reflect the similar feeding behaviors of these coral reef fishes. For instance, algae-farming damselfishes may also ingest prey items other than algae, such as zooplankton (Eurich et al. 2019) or other invertebrates (Letourneur et al. 1997). The functional roles of these seemingly important microbial taxa warrant further attention in order to understand the potential consequences on host metabolism and health.

Damselfish microbiomes were largely dominated by the family *Pasteurellaceae* in the phylum *Gammaproteobacteria*, with one ASV (b727) occurring in more than 80% of sampled fishes and representing almost 10% of the total detected sequences (Tables 1 and 3). Although this ASV currently represents an unknown species in the *Actinobacillus* genus, a 98% similar sequence has been retrieved from the intestines of surgeonfishes in Saudi Arabia (Miyake et al. 2016), suggesting that *Actinobacillus* are common members of reef fish microbiomes. Bacteria in the genus *Pasteurellaceae* have also been recorded in high abundances in adult damselfishes and cardinalfishes collected around Lizard Island, Australia (Parris et al. 2016), and they are deemed as common components of tropical planktivorous fish gut microbiomes (Egerton et al. 2018). The prevalence of *Pasteurellaceae* amongst the damselfishes in this study, as well as in other reef fishes, provides additional evidence that *Pasteurellaceae* are likely important members of coral reef-associated fish microbiomes.

Algae-farming damselfishes had more observed ASVs and larger core microbiomes than planktivorous species (Figs. 1 and 3), and these core microbiomes were specific to each host species (Fig. 3). For example, *P. wardi* and *P. moluccensis* microbiomes were dominated by different taxa of *Gammaproteobacteria*, while *D. perspicillatus* and *S. apicalis* had large *Bacteroidia* core communities but were dominated by *Flavobacteriaceae* and *Rikenellaceae*, respectively. Different species of algae-farming damselfishes consume different species of algae (Casey et al. 2014), and the large differences in their specialized microbiomes may reflect these narrow dietary preferences. Conversely, the small core microbiomes of the planktivorous damselfishes may reflect the high variation in consumed plankton of each species, suggesting these fishes have opportunistic feeding behaviors. These results, however, do not support the notion that fish with greater diet variability have more diverse microbiomes (Givens et al. 2015). In fact, the damselfish with narrow, algae-farming feeding behaviors tended to have the greatest diversity of intestinal bacteria, suggesting that the host-microbiome interactions may select for specialized bacteria that enhance the digestion and absorption of nutrients from specific algal diets. The richer microbiome of algae-farming fishes could also reflect the necessity of this trophic guild to be associated with a pool of symbionts that facilitate the breakdown of algal cellulose. We also acknowledge that some of the bacteria we retrieved from the damselfish intestine could have been associated with the food recently ingested by the fish and, therefore, not being part of the damselfish microbiome.

Evidence suggests a high degree of resource partitioning in fish communities, which is a key mechanism that facilitates the high diversity of coral reefs (Casey et al. 2019; Leray et al. 2019). The largely distinct microbiomes of each host species presented in this study may reflect the high degree of resource partitioning found in coral reef communities, whereby different species of damselfish may be consuming different size classes of zooplankton (Leray et al. 2019), farm different algal species (Casey et al. 2014), or occupy different trophic niches (Casey et al. 2019). The similarity between closely related host species and microbiomes, such as *P. wardi* and *P. moluccensis*, also demonstrates that phylogeny may influence the intestinal microbiomes of damselfishes (Sullam et al. 2012; Miyake et al. 2015; Neuman et al. 2016; Chiarello et al. 2018).

Interestingly, *Photobacterium damselae, Vibrio harveyi, Vibrio ponticus*, and other *Vibrio* sp. were prevalent amongst the damselfishes sampled in this study (Table 3). These bacteria represent potential pathogenic members of *Vibrionacaea* and have been detected in many fishes of aquaculture importance, including *Chromis punctipinnis* (Love et al. 1981), *Lutjanus argentimaculatus* (Reshma et al. 2018), *Seriola dumerili* (Nishiki et al. 2018), *Scophthalmus maximus* (Montes et al. 2003), *Sparus aurata* (Vera 1991), and *Solea senegalensis* (Terceti et al. 2016). Although identified as *Vibrio harveyi* in the GreenGenes database, GenBank revealed there was a high similarity of these sequences to other members of the *Harveyi* clade, such as *Vibrio owensii* (Nishiki et al. 2018). It is thought that there are up to 11 species of *Vibrio* belonging to this clade (Urbanczyk et al. 2013), most of which are pathogens of fish, shrimp, and coral (Thompson et al. 2004; Austin and Zhang 2006; Ushijima et al. 2012). Given the apparently healthy state of the sampled fishes and the high abundances of potentially pathogenic *Vibrionacaea* in the fish guts, we provide support to the idea that these organisms are natural components of healthy fish microbiomes and are opportunistic pathogens in fishes only under specific conditions (Rivas et al. 2013; Reshma et al. 2018). Future studies should also investigate the involvement of algae-farming damselfish in the spreading of pathogens across reef organisms. For instance, it has recently been reported that the seagrass pathogen *Labyrinthula* was present in the skeleton of a common coral species (Ricci et al. 2021) and probably infected the abundant endolithic algae living in the coral skeleton (Ricci et al. 2019; Iha et al. 2020; Tandon et al. 2022; Ricci et al. 2022). Thus, it is possible that damselfishes grazing near alive corals were the medium that allowed the pathogen *Labyrinthula* to infect the corals’ endolithic algae.

The facultative anaerobic bacterial classes *Bacteroidia, Clostridia*, and *Mollicutes* were generally in higher abundance in the mid and posterior intestinal regions than in the stomach (Fig. 4). Differences in microbiomes along the intestinal tract have been recorded in the rabbitfish *Siganus fuscescens* (Nielsen et al. 2017), with midgut communities more representative of the environmental sources and hindguts hosting a microbiome more specialized to anaerobic conditions and fermentation (Jones et al. 2018). The increase in *Bacteroidia, Clostridia*, and *Mollicutes* along the intestines may be due to some members of these bacterial classes being mutualistic components of the fish gastrointestinal microbiome. Some members of *Bacteroidia* are known to breakdown polysaccharides and metabolize the derived sugars (Xu et al. 2003), while members of *Clostridium* are known to metabolize cellulose (Liu et al. 2016). Our results confirm the increased prevalence of anaerobic bacteria in the hindgut of damselfishes, which probably consists of taxa responsible for the fermentation and metabolism of complex molecules before being absorbed by the host (Clements et al. 2014). We also note that *Actinobacillus* sp. that could breakdown cellulose via fermentation (Almqvist et al. 2016) were more abundant in the gut of algae-farming damselfish, suggesting that these bacteria could aid the digestion of fish in this trophic guild.

## Conclusions

In this study, we show that damselfishes have diverse intestinal microbial communities whereby the bacterial richness of a species reflects diet and trophic guild. We show that algae-farming damselfishes have richer bacterial alpha-diversity and core microbiomes, which may reflect the more specialized diets of this trophic guild. We also provide evidence that damselfish mid and posterior intestines have higher abundances of facultative anaerobic bacteria that are known to play important roles in fermentation and cellulose breakdown. These findings add to a growing body of literature that suggests that host fish feeding behavior has a strong influence on the composition of intestinal microbiomes.

## Supplementary Material

obac026_Supplemental_Files

## Data Availability

The Illumina MiSeq datasets for each damselfish species are available at the Sequence Read Archive (NCBI) repository under BioProject accession number PRJNA638998, https://www.ncbi.nlm.nih.gov/sra. Data and R-scripts used in this study are available at https://github.com/ChrisKav/WildDamselfishMicrobiomes.

## References

[bib1] Ainsworth TD , Krause L, Bridge T, Torda G, Raina JB, Zakrzewski M, Gates RD, Spalding HL, Smith C, Woolsey ES, Bourne DG et al. 2015. The coral core microbiome identifies rare bacterial taxa as ubiquitous endosymbionts. ISME J 9:2261–74. 25885563 10.1038/ismej.2015.39 PMC4579478

[bib2] Almqvist H , Pateraki C, Alexandri M, Koutinas AA. 2016. Succinic acid production by Actinobacillus succinogenes from batch fermentation of mixed sugars. J Ind Microbiol Biotechnol 43:1117–30. 27255975 10.1007/s10295-016-1787-x

[bib3] Austin B , Zhang XH. 2006. Vibrio harveyi: a significant pathogen of marine vertebrates and invertebrates. Lett Appl Microbiol 43:119–24. 16869892 10.1111/j.1472-765X.2006.01989.x

[bib4] Bates JM , Mittge E, Kuhlman JA, Baden KN, Cheeseman SE, Guillemin K. 2006. Distinct signals from the microbiota promote different aspects of zebrafish gut differentiation. Dev Biol 297:374–86. 16781702 10.1016/j.ydbio.2006.05.006

[bib5] Bates JM , Akerlund J, Mittge E, Guillemin K. 2007. Intestinal alkaline phosphatase detoxifies lipopolysaccharide and prevents inflammation in zebrafish in response to the gut microbiota. Cell Host Microbe 2:371–82. 18078689 10.1016/j.chom.2007.10.010 PMC2730374

[bib6] Blanchette A , Ely T, Zeko A, Sura SA, Turba R, Fong P. 2019. Damselfish Stegastes nigricans increase algal growth within their territories on shallow coral reefs via enhanced nutrient supplies. J Experim Mar Biol and Ecol 513:21–6.

[bib7] Bokulich NA , Kaehler BD, Rideout JR, Dillon M, Bolyen E, Knight R, Huttley GA, Caporaso JG. 2018. Optimizing taxonomic classification of marker-gene amplicon sequences with QIIME 2’s q2-feature-classifier plugin. Microbiome 6:1–17. 29773078 10.1186/s40168-018-0470-z PMC5956843

[bib8] Bolnick DI , Snowberg LK, Hirsch PE, Lauber CL, Org E, Parks B, Lusis AJ, Knight R, Caporaso JG, Svanback R. 2014. Individual diet has sex-dependent effects on vertebrate gut microbiota. Nat Commun 5:1–13. 10.1038/ncomms5500 PMC4279269 25072318

[bib9] Bolyen E , Rideout JR, Dillon MR, Bokulich NA, Abnet CC, Ghalith GAA, Alexander H, Alm EJ, Arumugam M, Asnicar F et al. 2018. QIIME 2: Reproducible, interactive, scalable, and extensible microbiome data science. PeerJ Preprints. 10.1038/s41587-019-0209-9 PMC7015180 31341288

[bib10] Callahan BJ , Mcmurdie PJ, Rosen MJ, Han AW, Johnson A, Holmes SP. 2016. DADA2: high-resolution sample inference from Illumina amplicon data. Nat Methods 13:581–3. 27214047 10.1038/nmeth.3869 PMC4927377

[bib11] Campbell MA , Robertson DR, Vargas MI, GR Allen, McMillan WO. 2018. Multilocus molecular systematics of the circumtropical reef-fish genus Abudefduf (*Pomacentridae*): history, geography and ecology of speciation. PeerJ 6:e5357. 30128183 10.7717/peerj.5357 PMC6097498

[bib12] Casey J , Choat JH, Connolly SR. 2015. Coupled dynamics of territorial damselfishes and juvenile corals on the reef crest. Coral Reefs 34:1–11.

[bib13] Casey JM , Ainsworth TD, Choat JH, Connolly SR. 2014. Farming behavior of reef fishes increases the prevalence of coral disease associated microbes and black band disease. Proc Royal Soc B: Biol Sci 281:20141032. 10.1098/rspb.2014.1032 PMC4083805 24966320

[bib14] Casey JM , Connolly SR, Ainsworth TD. 2015. Coral transplantation triggers shift in microbiome and promotion of coral disease associated potential pathogens. Sci Rep 5:1–11. 10.1038/srep11903 PMC4491727 26144865

[bib15] Casey JM , Meyer CP, Morat F, Brandl SJ, Planes S, Parravicini V. 2019. Reconstructing hyperdiverse food webs: gut content metabarcoding as a tool to disentangle trophic interactions on coral reefs. Meth in Ecol and Evol 10:1157–70.

[bib16] Ceccarelli D. 2007. Modification of benthic communities by territorial damselfish: a multi-species comparison. Coral reefs 26:853–66.

[bib17] Ceccarelli DM , Jones GP, McCook LJ. 2005. Effects of territorial damselfish on an algal-dominated coastal coral reef. Coral Reefs 24:606–20.

[bib18] Cheesman SE , Neal JT, Mittge E, Seredick BM, Guillemin K. 2011. Epithelial cell proliferation in the developing zebrafish intestine is regulated by the Wnt pathway and microbial signaling via Myd88. Proc Nat Acad of Sci 108:4570–7. 20921418 10.1073/pnas.1000072107 PMC3063593

[bib19] Chen H , Boutros PC. 2011. VennDiagram: a package for the generation of highly-customizable Venn and Euler diagrams in R. BMC Bioinformatics 12:1–7. 21269502 10.1186/1471-2105-12-35 PMC3041657

[bib20] Chiarello M , Auguet JC, Bettarel Y, Bouvier C, Claverie T, Graham NA, Rieuvilleneuve F, Sucre E, Bouvier T, Villeger S. 2018. Skin microbiome of coral reef fish is highly variable and driven by host phylogeny and diet. Microbiome 6:1–14. 30143055 10.1186/s40168-018-0530-4 PMC6109317

[bib21] Choat J. 1991. The biology of herbivorous fishes on coral reefs. In: Sale PF, (ed.) The Ecology of Fishes on Coral Reefs. San Diego: Academic Press.

[bib22] Clements KD , Angert ER, Montgomery WL, Choat JH. 2014. Intestinal Microbiota in Fishes: What's Known and What's Not. Wiley Online Library. 10.1111/mec.12699 24612310

[bib23] Cooper WJ , Smith LL, Westneat MW. 2009. Exploring the radiation of a diverse reef fish family: phylogenetics of the damselfishes (*Pomacentridae*), with new classifications based on molecular analyses of all genera. Mol Phylogenet and Evol 52:1–16. 10.1016/j.ympev.2008.12.010 19135160

[bib24] Desai AR , Links MG, Collins SA, Mansfield GS, Drew MD, Kessel AG, Hill JE. 2012. Effects of plant-based diets on the distal gut microbiome of rainbow trout (*Oncorhynchus mykiss*). Aquaculture 350:134–42.

[bib25] Egerton S , Culloty S, Whooley J, Stanton C, Ross RP. 2018. The gut microbiota of marine fish. Front microbiol 9:873. 29780377 10.3389/fmicb.2018.00873 PMC5946678

[bib26] Emslie MJ , Logan M, Ceccarelli DM, Cheal AJ, Hoey AS, Miller I, Sweatman HP. 2012. Regional-scale variation in the distribution and abundance of farming damselfishes on Australia's Great Barrier Reef. Marine Biol 159:1293–304.

[bib27] Emslie MJ , Logan M, Cheal AJ. 2019. The distribution of planktivorous damselfishes (*Pomacentridae*) on the Great Barrier Reef and the relative influences of habitat and predation. Diversity 11:33.

[bib28] Eurich J , Mccormick MI, Jones GP. 2018. Habitat selection and aggression as determinants of fine-scale partitioning of coral reef zones in a guild of territorial damselfishes. Mar Ecol Progr Ser 587:201–15.

[bib29] Eurich J , Matley JK, Baker R, Mccormick MI, Jones GP. 2019. Stable isotope analysis reveals trophic diversity and partitioning in territorial damselfishes on a low-latitude coral reef. Marine Biol 166:1–14.

[bib30] Eurich JG , Shomaker SM, McCormick MI, Jones GP. 2018. Experimental evaluation of the effect of a territorial damselfish on foraging behavior of roving herbivores on coral reefs. J of Experim Marine Biol and Ecol 506:155–62.

[bib31] Eurich JG , McCormick MI, Jones GP. 2018. Direct and indirect effects of interspecific competition in a highly partitioned guild of reef fishes. Ecosphere 9:e02389.

[bib32] Foster KR , Schluter J, Coyte KZ, Nahoum SR. 2017. The evolution of the host microbiome as an ecosystem on a leash. Nat 548:43–51. 10.1038/nature23292 PMC5749636 28770836

[bib33] Galindo-Villegas J , Moreno DG, Oliveira SD, Mulero V. 2012. Regulation of immunity and disease resistance by commensal microbes and chromatin modifications during zebrafish development. Proc Nati Acad Sci 109:E2605–14. 10.1073/pnas.1209920109 PMC3465450 22949679

[bib34] Gibson R , McCook LJ, Ceccarelli DM. 2001. Territorial damselfishes as determinants of the structure of benthic communities on coral reefs. Oceanogra and Marine Biol: An Ann Rev 39:355–89.

[bib35] Givens CE , Ransom B, Bano N, Hollibaugh JT. 2015. Comparison of the gut microbiomes of 12 bony fish and 3 shark species. Marine Ecol Progress Series 518:209–23.

[bib36] Gochfeld DJ. 2010. Territorial damselfishes facilitate survival of corals by providing an associational defense against predators. Marine Ecol Progress Series 398:137–48.

[bib37] Hagi T , Tanaka D, Iwamura Y, Hoshino T. 2004. Diversity and seasonal changes in lactic acid bacteria in the intestinal tract of cultured freshwater fish. Aquaculture 234:335–46.

[bib38] Hata H , Kato M. 2004. Monoculture and mixed-species algal farms on a coral reef are maintained through intensive and extensive management by damselfishes. J of Experim Marine Biol and Ecol 313:285–96.

[bib39] He S , Wu Z, Liu Y, Wu N, Tao Y, Xu L, Zhou Z, Yao B, Ringo E. 2013. Effects of dietary 60 g kg− 1 dried distiller's grains in least-cost practical diets on production and gut allochthonous bacterial composition of cage-cultured fish: comparison among fish species with different natural food habits. Aquacul Nutr 19:765–72.

[bib40] Iha C , Dougan KE, Varela JA, Avila V, Jackson CJ, Bogaert KA, Chen Y, Judd LM, Wick R, Holt KE et al. 2020. Genomic adaptations to an endolithic lifestyle in the coral-associated alga Ostreobium. Curr Biol 31:1393–1402. doi: 10.1101/2020.07.21.211367. bioRxiv 10.1016/j.cub.2021.01.018 33548192

[bib41] Ingerslev H-C , Jorgensen LG, Strube ML, Larsen N, Dalsgaard I, Boye M, Madsen L. 2014. The development of the gut microbiota in rainbow trout (*Oncorhynchus mykiss*) is affected by first feeding and diet type. Aquaculture 424:24–34.

[bib42] Jones J , DiBattista JD, Stat M, Bunce M, Boyce MC, Fairclough DV, Travers MJ, Huggett MJ. 2018. The microbiome of the gastrointestinal tract of a range-shifting marine herbivorous fish. Front Microbiol 9:2000. 30210475 10.3389/fmicb.2018.02000 PMC6121097

[bib43] Kasumyan A. 2009. Acoustic signaling in fish. J of Ichthyol 49:963–1020.

[bib44] Klumpp D , Mckinnon AD, Daniel P. 1987. Damselfish territories: zones of high productivity on coral reefs. Marine ecology progress series. Oldendorf 40:41–51.

[bib45] Lane DJ , Pace B, Olsen GJ, Stahl DA, Sogin ML, Pace NR. 1985. Rapid determination of 16S ribosomal RNA sequences for phylogenetic analyses. Proc Nat Acad Sci 82:6955–9. 2413450 10.1073/pnas.82.20.6955 PMC391288

[bib46] Leray M , Alldredge AL, Yang JY, Meyer CP, Holbrook SJ, Schmitt RJ, Knowlton N, Brooks AJ. 2019. Dietary partitioning promotes the coexistence of planktivorous species on coral reefs. Mol Ecol 28:2694–710. 30933383 10.1111/mec.15090 PMC6852152

[bib47] Letourneur Y , Galzin R, Harmelin MV. 1997. Temporal variations in the diet of the damselfish Stegastes nigricans (*Lacepede*) on a Reunion fringing reef. J of Experim Marine Biol and Ecol 217:1–18.

[bib48] Letunic I , Bork P. 2016. Interactive tree of life (iTOL) v3: an online tool for the display and annotation of phylogenetic and other trees. Nucleic Acids Res 44:W242–5. 27095192 10.1093/nar/gkw290 PMC4987883

[bib49] Li J , Ni J, Li J, Wang C, Li X, Wu S, Zhang T, Yu Y, Yan Q. 2014. Comparative study on gastrointestinal microbiota of eight fish species with different feeding habits. J of Appl Microbiol 117:1750–60. 25294734 10.1111/jam.12663

[bib50] Li X , Yan Q, Ringo E, Wu X, He Y, Yang D. 2016. The influence of weight and gender on intestinal bacterial community of wild largemouth bronze gudgeon (*Coreius guichenoti*, 1874). BMC Microbiol 16:1–8. 27549138 10.1186/s12866-016-0809-1 PMC4994167

[bib51] Liu H , Guo X, Gooneratne R, Lai R, Zeng C, Zhan F, Wang W. 2016. The gut microbiome and degradation enzyme activity of wild freshwater fishes influenced by their trophic levels. Sci Rep 6:1–12. 27072196 10.1038/srep24340 PMC4829839

[bib52] Love M , Fisher DT, Hose JE, Farmer JJ, Hickman FW, Fanning GR. 1981. Vibrio damsela, a marine bacterium, causes skin ulcers on the damselfish Chromis punctipinnis. Sci 214:1139–40. 10.1126/science.214.4525.1139 17755898

[bib53] Ludwig W. 2007. Nucleic acid techniques in bacterial systematics and identification. Int J Food Microbiol 120:225–36. 17961780 10.1016/j.ijfoodmicro.2007.06.023

[bib54] Martin-Antonio B , Manchado M, Infante C, Zerolo R, Labella A, Alonso C, Borrego JJ. 2007. Intestinal microbiota variation in Senegalese sole (*Solea senegalensis*) under different feeding regimes. Aquacul Res 38:1213–22.

[bib55] McMurdie PJ. 2018. Normalization of microbiome profiling data. In Microbiome Analysis, pp. 143–68, New York: Springer. 10.1007/978-1-4939-8728-3_10 30298253

[bib56] McMurdie PJ , Holmes S. 2013. phyloseq: an R package for reproducible interactive analysis and graphics of microbiome census data. PloS One 8:e61217. 23630581 10.1371/journal.pone.0061217 PMC3632530

[bib57] McMurdie PJ , Holmes S. 2014. Waste not, want not: why rarefying microbiome data is inadmissible. PLoS Comput Biol 10:e1003531. 24699258 10.1371/journal.pcbi.1003531 PMC3974642

[bib58] Miyake S , Ngugi DK, Stingl U. 2015. Diet strongly influences the gut microbiota of surgeonfishes. Mol Ecol 24:656–72. 25533191 10.1111/mec.13050

[bib59] Miyake S , Ngugi DK, Stingl U. 2016. Phylogenetic diversity, distribution, and cophylogeny of giant bacteria (*Epulopiscium*) with their surgeonfish hosts in the Red Sea. Front Microbiol 7:285. 27014209 10.3389/fmicb.2016.00285 PMC4789555

[bib60] Montes M , Farto R, Perez MJ, Nieto TP, Larsen JL, Christensen H. 2003. Characterization of Vibrio strains isolated from turbot (*Scophthalmus maximus*) culture by phenotypic analysis, ribotyping and 16S rRNA gene sequence comparison. J Appl Microbiol 95:693–703. 12969281 10.1046/j.1365-2672.2003.02028.x

[bib61] Nayak SK. 2010. Role of gastrointestinal microbiota in fish. Aquacul Res 41:1553–73.

[bib62] Neish AS. 2009. Microbes in gastrointestinal health and disease. Gastroenterol 136:65–80. 10.1053/j.gastro.2008.10.080 PMC2892787 19026645

[bib63] Neuman C , Hatje E, Zarkasi KZ, Smullen R, Bowman JP, Katouli M. 2016. The effect of diet and environmental temperature on the faecal microbiota of farmed T asmanian A tlantic S almon (*S almo salar L.*). Aquacul Res 47:660–72.

[bib64] Ngugi DK , Miyake S, Cahill M, Sting U. 2017. Genomic diversification of giant enteric symbionts reflects host dietary lifestyles. Proc Nat Acad Sci 114:E7592–601. 28835538 10.1073/pnas.1703070114 PMC5594648

[bib65] Nielsen S , Walburn JW, Verges A, Thomas T, Egan S. 2017. Microbiome patterns across the gastrointestinal tract of the rabbitfish Siganus fuscescens. PeerJ 5:e3317. 28533966 10.7717/peerj.3317 PMC5437856

[bib66] Nishiki I , Minami T, Murakami A, Hoai TD, Fujiwara A. 2018. Multilocus sequence analysis of Vibrionaceae isolated from farmed amberjack and the development of a multiplex PCR assay for the detection of pathogenic species. J Fish Dis 41:1295–301. 10.1111/jfd.12823 29882274

[bib67] Oksanen J , Blanchet FG, Kindt R, Legendre P, Minchin PR, O'hara RB, Simpson GL, Solymos P, Stevens MH, Wagner H et al. 2017. Package “vegan”: Community ecology package. R Package Version 2:5–6.

[bib68] Parris DJ , Brooker RM, Morgan MA, Dixson DL, Stewart FJ. 2016. Whole gut microbiome composition of damselfish and cardinalfish before and after reef settlement. PeerJ 4:e2412. 27635360 10.7717/peerj.2412 PMC5012416

[bib69] Pedregosa F , Varoquax G, Gramfort A, Michel V, Thirion B, Grisel O, Blondel M, Prettenhofer P, Weiss R, Dubourg V et al. 2011. Scikit-learn: machine learning in Python. J Mach Learn Res 12:2825–30.

[bib70] Price MN , Dehal PS, Arkin AP. 2010. FastTree 2–approximately maximum-likelihood trees for large alignments. PloS one 5:e9490. 20224823 10.1371/journal.pone.0009490 PMC2835736

[bib71] Randazzo Eisemann Á , Munoz JL, Mcfield M, Myton J, Gonzalez JE. 2019. The effect of algal-gardening damselfish on the resilience of the Mesoamerican Reef. Front Marine Sci 6:414.

[bib72] Rawls JF , Samuel BS, Gordon JI. 2004. Gnotobiotic zebrafish reveal evolutionarily conserved responses to the gut microbiota. Proc Nat Aca Sci 101:4596–601. 10.1073/pnas.0400706101 PMC384792 15070763

[bib73] Ray A , Ghosh k, Ringo E. 2012. Enzyme-producing bacteria isolated from fish gut: a review. Aquacul Nut 18:465–92.

[bib74] Reshma K , Sumithra TG, Anusree VN, Raju S, Kishor TG, SreenathKR Sanil NK. 2018. An insight into the gut microbiology of wild-caught mangrove red snapper, lutjanus argentimaculatus (forsskal, 1775). Aquaculture 497:320–30.

[bib75] Ricci F , Marcelino VR, Blackall LL, Kuhl M, Medina M, Verbruggen H. 2019. Beneath the surface: community assembly and functions of the coral skeleton microbiome. Microbiome 7:1–10. 31831078 10.1186/s40168-019-0762-y PMC6909473

[bib76] Ricci F , Fordyce A, Leggat W, Blackall LL, Ainsworth T, Verbruggen H. 2021. Multiple techniques point to oxygenic phototrophs dominating the Isopora palifera skeletal microbiome. Coral Reefs 40:275–82.

[bib77] Ricci F , Tandon K, Mobhammer M, Cho EH, Blackall LL, Kuhl M, Verbruggen H. 2022. Fine-scale mapping of physicochemical and microbial landscapes clarifies the spatial structure of the coral skeleton microbiome. ResearchSquare. doi:10.21203/rs.3.rs-1735748/v1.

[bib78] Ricci F , Tandon K, Black JR, Cao KA, Blackall LL, Verbruggen H. 2022. Host traits and phylogeny contribute to shaping coral-bacterial symbioses. Msystems 7:e00044–22. 10.1128/msystems.00044-22 PMC9045482 35253476

[bib79] Ringø E , Sperstad S, Myklebust R, Refstie S, Krogdahl A. 2006. Characterization of the microbiota associated with intestine of Atlantic cod (*Gadus morhua L.*): the effect of fish meal, standard soybean meal, and a bioprocessed soybean meal. Aquaculture 261:829–41.

[bib80] Rivas AJ , Lemos ML, Osorio CR. 2013. Photobacterium damselae subsp. damselae, a bacterium pathogenic for marine animals and humans. Front Microbiol 4:283. 24093021 10.3389/fmicb.2013.00283 PMC3782699

[bib81] Stephens WZ , Burns AR, Stagaman K, Wong S, Rawls JF, Guillemin K, Bohannan BJ. 2016. The composition of the zebrafish intestinal microbial community varies across development. ISME J 10:644–54. 26339860 10.1038/ismej.2015.140 PMC4817687

[bib82] Sullam KE , Essinger SD, Lozupone CA, Connor MP, Rosen GL, Knight R, Kilham SS, Russel JA. 2012. Environmental and ecological factors that shape the gut bacterial communities of fish: a meta-analysis. Mol Ecol 21:3363–78. 22486918 10.1111/j.1365-294X.2012.05552.x PMC3882143

[bib83] Tandon K , Pasella MM, Iha C, Ricci F, HU J, Kelly CK, Medina M, Kuhl M, Verbruggen H. 2022. Every refuge has its price: ostreobium as a model for understanding how algae can live in rock and stay in business. Semin Cell Dev Biol Elsevier. 10.1016/j.semcdb.2022.03.010 35341677

[bib84] Tebbett SB , Chase TJ, Bellwood DR. 2020. Farming damselfishes shape algal turf sediment dynamics on coral reefs. Mar Environ Res 160:104988. 32907726 10.1016/j.marenvres.2020.104988

[bib85] Terceti MS , Ogut H, Osorio CR. 2016. Photobacterium damselae subsp. damselae, an emerging fish pathogen in the Black Sea: evidence of a multiclonal origin. Appl Environ Microbiol 82:3736–45. 27084008 10.1128/AEM.00781-16 PMC4907178

[bib86] Thompson FL , Lida T, Swings J. 2004. Biodiversity of vibrios. Microbiol Mol Biol Rev 68:403–31. 15353563 10.1128/MMBR.68.3.403-431.2004 PMC515257

[bib87] Urbanczyk H , Ogura Y, Hayashi T. 2013. Taxonomic revision of Harveyi clade bacteria (family *Vibrionaceae*) based on analysis of whole genome sequences. Internat J of Sys Evol Microbiol 63:2742–51. 10.1099/ijs.0.051110-0 23710045

[bib88] Ushijima B , Smith A, Aeby GS, Callahan SM. 2012. Vibrio owensii induces the tissue loss disease Montipora white syndrome in the Hawaiian reef coral Montipora capitata. PLoS One 7:e46717. 23056419 10.1371/journal.pone.0046717 PMC3466290

[bib89] Vera P 1991. First isolation of Vibrio damsela from sea bream (*Sparus aurata*). Bull. Eur. Ass. Fish Pathol. 11:112.

[bib90] Wang AR et al. 2018. Progress in fish gastrointestinal microbiota research. Rev Aquacul 10:626–40.

[bib91] Wang Y , Naumann U, ST Wright, Warton DI. 2012. mvabund–an R package for model-based analysis of multivariate abundance data. Meth Ecol Evol 3:471–4.

[bib92] Weimann SR , Black AN, Leese J, Richter ML, Itzkowitz M, Burger RM. 2018. Territorial vocalization in sympatric damselfish: acoustic characteristics and intruder discrimination. Bioacoustics 27:87–102.

[bib93] Xu J , Bjursell MK, Himrod J, Deng S, Carmichael LK, Chiang HC, Hooper LV, Gordon JI. 2003. A genomic view of the human-Bacteroides thetaiotaomicron symbiosis. Sci 299:2074–6. 10.1126/science.1080029 12663928

[bib94] Zhang X , Wu H, Li Z, Li Y, Wang S, Zhu D, Wen X, Li S. 2018. Effects of dietary supplementation of Ulva pertusa and non-starch polysaccharide enzymes on gut microbiota of Siganus canaliculatus. J of Oceanol and Limnol 36:438–49.

