# Supplementary material for: Intestinal Microbiome Richness of Coral Reef Damselfishes (Actinopterygii: Pomacentridae)
Source: Integr Org Biol. 2022 Sep 16;4(1):obac026. doi: 10.1093/iob/obac026 (PMC9486986; doi:10.1093/iob/obac026)
Supplement: obac026_Supplemental_Files [file obac026_supplemental_files.zip › Supplementary_Figure_1_v3.pdf]

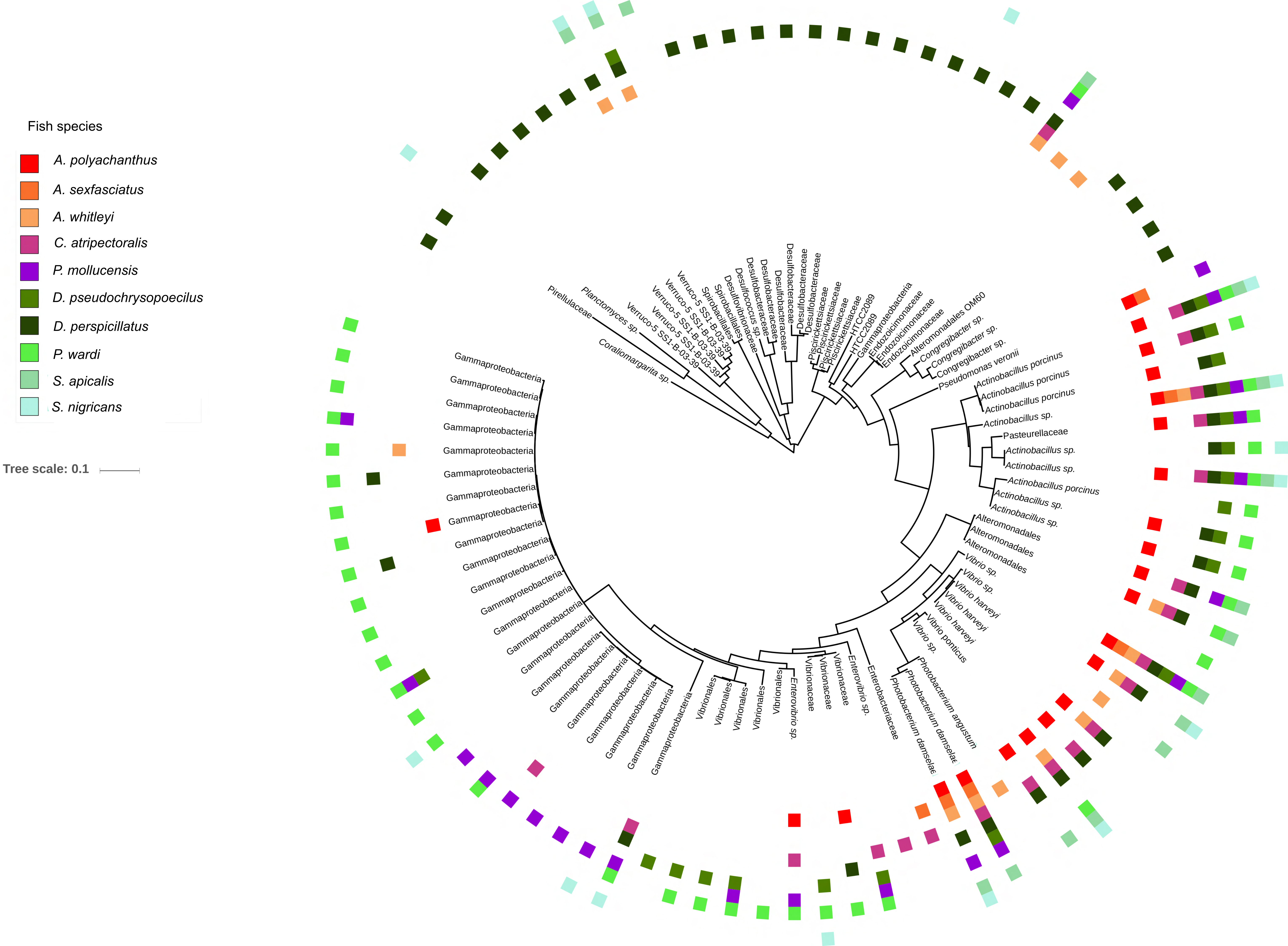

Fish species
A. polyachanthus
A. sexfasciatus
A. whitleyi
C. atripectoralis
P. mollucensis
D. pseudochrysopoecilus
D. perspicillatus
P. wardi
S. apicalis
S. nigricans
Tree scale: 0.1
