# Supplementary material for: Intestinal Microbiome Richness of Coral Reef Damselfishes (Actinopterygii: Pomacentridae)
Source: Integr Org Biol. 2022 Sep 16;4(1):obac026. doi: 10.1093/iob/obac026 (PMC9486986; doi:10.1093/iob/obac026)
Supplement: obac026_Supplemental_Files [file obac026_supplemental_files.zip › Supplementary_Figure_3_v3.pdf]

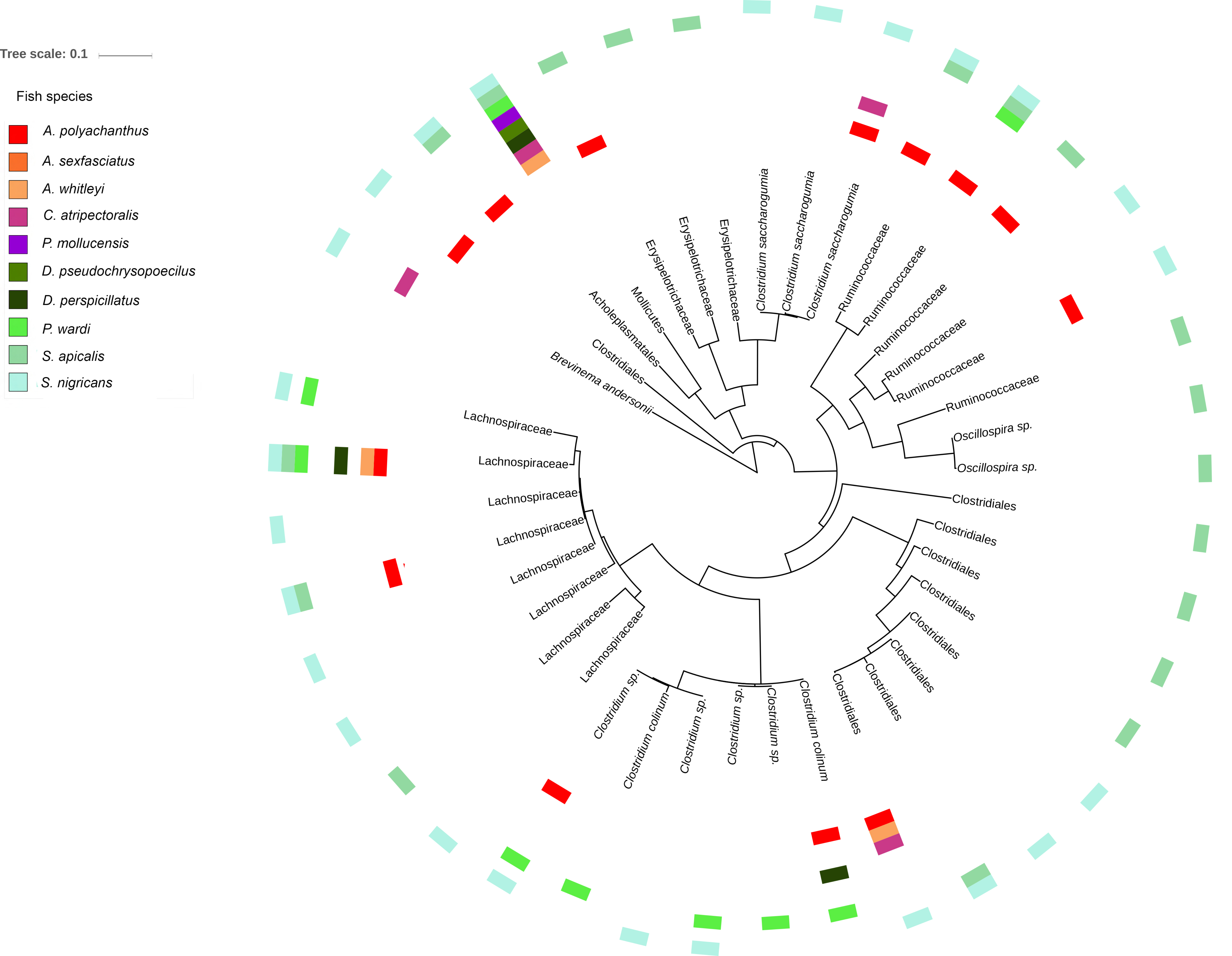

Tree scale: 0.1
Fish species
A. polyachanthus
A. sexfasciatus
A. whitleyi
C. atripectoralis
P. mollucensis
D. pseudochrysopoecilus
D. perspicillatus
P. wardi
S. apicalis
S. nigricans
Brevinema andersonii
Clostridiales
Acholeplasmatales
Mollicutes
Erysipelotrichaceae
Erysipelotrichaceae
Erysipelotrichaceae
Clostridium saccharogumia
Clostridium saccharogumia
Clostridium saccharogumia
Ruminococcaceae
Ruminococcaceae
Ruminococcaceae
Ruminococcaceae
Ruminococcaceae
Ruminococcaceae
Oscillospira sp.
Oscillospira sp.
Clostridiales
Clostridiales
Clostridiales
Clostridiales
Clostridiales
Clostridiales
Clostridiales
Clostridiales
Clostridium colinum
Clostridium sp.
Clostridium sp.
Clostridium sp.
Clostridium colinum
Clostridium sp.
Lachnospiraceae
Lachnospiraceae
Lachnospiraceae
Lachnospiraceae
Lachnospiraceae
Lachnospiraceae
Lachnospiraceae
Lachnospiraceae
