# Supplementary material for: Intestinal Microbiome Richness of Coral Reef Damselfishes (Actinopterygii: Pomacentridae)
Source: Integr Org Biol. 2022 Sep 16;4(1):obac026. doi: 10.1093/iob/obac026 (PMC9486986; doi:10.1093/iob/obac026)
Supplement: obac026_Supplemental_Files [file obac026_supplemental_files.zip › Supplementary_Tables.docx]

Supplementary Table 1: ANOVA table summarising multivariate GLM model of gut location nested within fish species. Results based on 999 simulations.

|  | Dfresidual | Dfdiff | LRT | *p* |
| --- | --- | --- | --- | --- |
| Gut Location | 115 | 3 | 206.8338 | 0.001 |
| Trophic guild | 114 | 1 | 304.6250 | 0.001 |
| Gut x Trophic | 111 | 3 | 152.0174 | 0.001 |

Supplementary Table 2: ANOVA table summarising multivariate GLM model of trophic guild and fish species as factors. Results based on 999 simulations.

|  | Dfresidual | Dfdiff | LRT | *p* |
| --- | --- | --- | --- | --- |
| Trophic guild | 1 | 19 | -0.0205 | 0.001 |
| Fish Species | 9 | 20 | 14213.000 | 0.001 |
| Trophic x Spec | 1 | 20 | -0.0229 | 1.000 |
