# Supplementary figures and images for: Intestinal Microbiome Richness of Coral Reef Damselfishes (Actinopterygii: Pomacentridae)
Source: Integr Org Biol. 2022 Sep 16;4(1):obac026. doi: 10.1093/iob/obac026 (PMC9486986; doi:10.1093/iob/obac026)

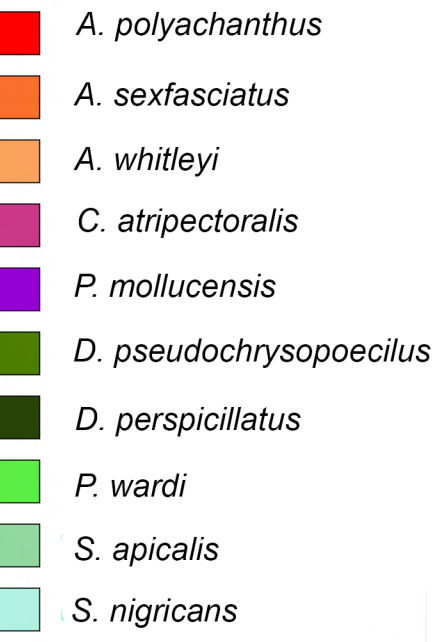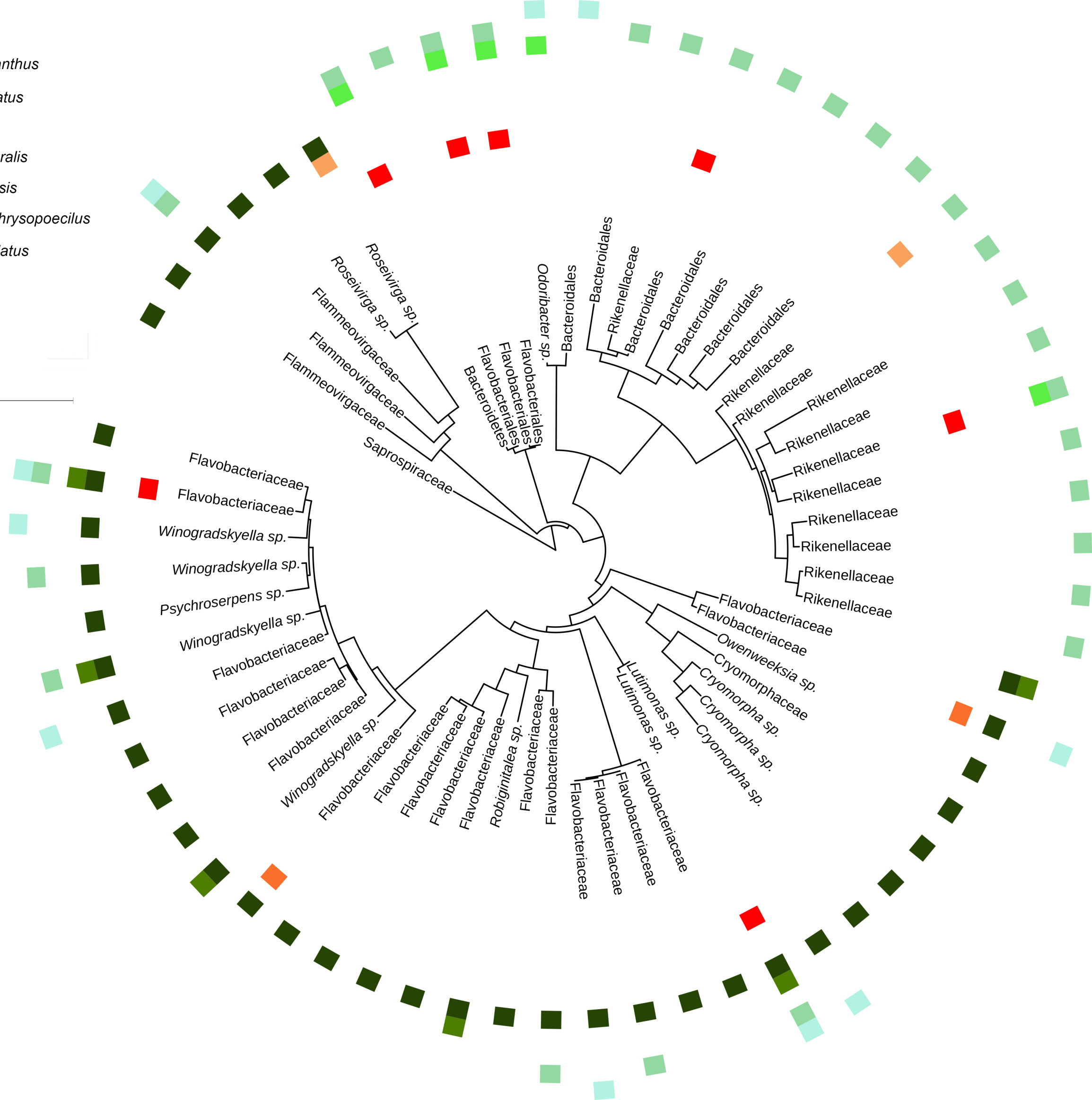

Supplement: obac026_Supplemental_Files [file obac026_supplemental_files.zip › Supplementary_Figure_2_v3.pdf]
